# Risk factors for conversion to total hip arthroplasty after acetabular fractures

**DOI:** 10.1007/s00068-024-02621-9

**Published:** 2024-08-21

**Authors:** Colin Christiaans, Sepp Hoogmoet, Wim Rijnen, Vincent Stirler, Erik Hermans

**Affiliations:** 1 Department of Trauma surgery, Radboudumc, Nijmegen, The Netherlands; 2 Department of Orthopaedics, Radboudumc, Nijmegen, The Netherlands; 3 Military Health Organisation, Ministery of defence, Kromhout Kazerne, Herculeslaan 1, Utrecht, 3584 AB the Netherlands

**Keywords:** Acetabular fracture, Total hip arthroplasty, Risk factors, Fracture classification

## Abstract

**Objectives:**

To identify acetabular fracture patterns classified according to Letournel that are at risk of conversion to total hip arthroplasty (THA).

**Design:**

A retrospective cohort study.

**Setting:**

A Level-I trauma center.

**Patients/ Participants:**

Patients with an acetabular fracture, classified according to Letournel who were treated with ORIF (*n* = 280).

**Interventions:**

Various surgical treatments for acetabular fractures.

**Main outcome measure:**

The primary outcome was the rate of conversion to total hip arthroplasty.

**Results:**

In this study, an overall conversion rate to THA of 13.9% within 2.2 years after initial surgery was found. Multivariate analysis revealed that several factors, namely, T-shaped fracture patterns (OR: 7.5, 95% CI 1.9–28.8, *p* = 0.003) and residual displacement (> 2 mm) (OR: 3.7, 95% CI 1.6–8.5, *p* = 0.002) are associated with an increased risk of conversion to THA. Furthermore, the risk of conversion to THA increases with 4.7% per gained year of age (OR: 1.047, 95% CI 1.0-1.1, *p* = 0,001). Other fracture patterns classified according to Letournel were not found to be independent risk factors.

**Conclusion:**

The presence of T-shaped fracture patterns is found to be an independent risk factor for conversion to THA. Furthermore, age and degree of reduction are found to be independent risk factors, which is in line with existing literature. These finding should be taken into account when treating patients with T-shaped acetabular fractures.

**Level of evidence:**

Prognostic study level III. See Instructions for Authors for a complete description of levels of evidence.

## Introduction

Acetabular fracture treatment remains a great challenge for surgeons. Operative treatment is necessary to restore continuity of the hip joint to preserve hip function and prevent coxarthrosis. Conservative treatment can be chosen in case of no displacement and for individual patients who are not fit for surgery because of high age or comorbidities.

Open reduction and internal fixation (ORIF) is currently considered to be the standard operative treatment procedure in patients with acetabular fractures [1–6]. However, despite improvements in the operative treatment, development of posttraumatic osteoarthritis of the hip joint is seen in 12–57% [6–9]. Posttraumatic osteoarthritis of the hip joint results in impaired hip joint function, chronic musculoskeletal pain, and results in a high rate of conversion to total hip arthroplasty (THA) (8.5–30.9%) [1, 3, 10–12]. In contrast to primary THA for osteoarthritis, conversion to THA after initial operative treatment for acetabular fractures could be challenging and carry a higher risk of complications, such as aseptic loosening, infection, dislocation and presence of heterotopic ossification. [7, 13–17].

Understanding the risk factors that contribute to conversion to total hip arthroplasty (THA) following acetabular fractures is crucial. This knowledge is essential for predicting poor outcomes after ORIF and for considering appropriate treatment alternatives. Multiple studies report different risk factors for conversion to THA after acetabular fractures, such as malreduction with residual steps and gaps, cartilage damage to the femoral head and/or acetabulum or patient age > 40 years [3, 10–12, 18]. A review by De Bellis et al. [19] reported that acute primary THA as initial treatment for acetabular fractures can be successful in elderly patients or patients with extensive osteoporosis, combined acetabular and femoral neck fractures or pathological fractures. Other recent studies suggest that initial treatment with ORIF combined with THA for specific acetabular fractures can be a successful treatment [20–22].

Although multiple studies report risk factors for conversion to THA after acetabular fractures, there is limited evidence if specific fracture patterns are prone to conversion to THA [11]. Therefore, the aim of this study is to specify the risk of conversion to THA in relation to fracture patterns classified by Letournel [23].

## Materials and methods

After approval was obtained from the institutional research board, the electronic patient database was searched for patients with acetabular fractures treated between 2014 and 2021. All patients were treated in a single level-I trauma centre in the Netherlands. All patients included were treated with ORIF initially. ORIF was done according to the discretion of the treating surgeon to provide the best fixation possible. All adult patients (> 16 years) with elementary or associated acetabular fractures according to fracture classification according to Letournel [23] were included. Exclusion criteria were death after trauma, non-operative treatment or lost to follow-up. If patients received initial treatment at our hospital but follow-up and further treatment was continued elsewhere, this was considered as lost to follow-up. Medical records were reviewed to obtain standard demographic characteristics such as age at time of trauma, gender, time from trauma to initial surgery in days, conversion to THA, time from initial surgery to conversion to THA in months and follow-up in months were obtained as well. To minimize recall bias, a chart review was performed. If information was lacking, a patient was excluded. *According to our standard protocol*,* all patients received a pre-op X-ray and CT scan as well as an inter-operative or post-operative CT scan within 48 h after surgery.* Radiological images of fractures at presentation to the emergency department were classified according to Letournel [23]. Residual displacement after reduction was measured in millimetres using intraoperative and/or postoperative CT-scan images. Residual displacement consisted of steps and gaps and was measured along the articular surface of the weight-bearing part of the acetabular dome. Residual displacement of < 2 mm (for both steps and gaps) was considered anatomical reduction. Fracture classification according to Letournel and measuring the degree of reduction was done retrospectively by two senior authors (VS and EH), with extensive experience in the treatment of acetabular fractures, using CT-scan images (consisting of axial, sagittal and coronal plane views).

**Fig. 1:**
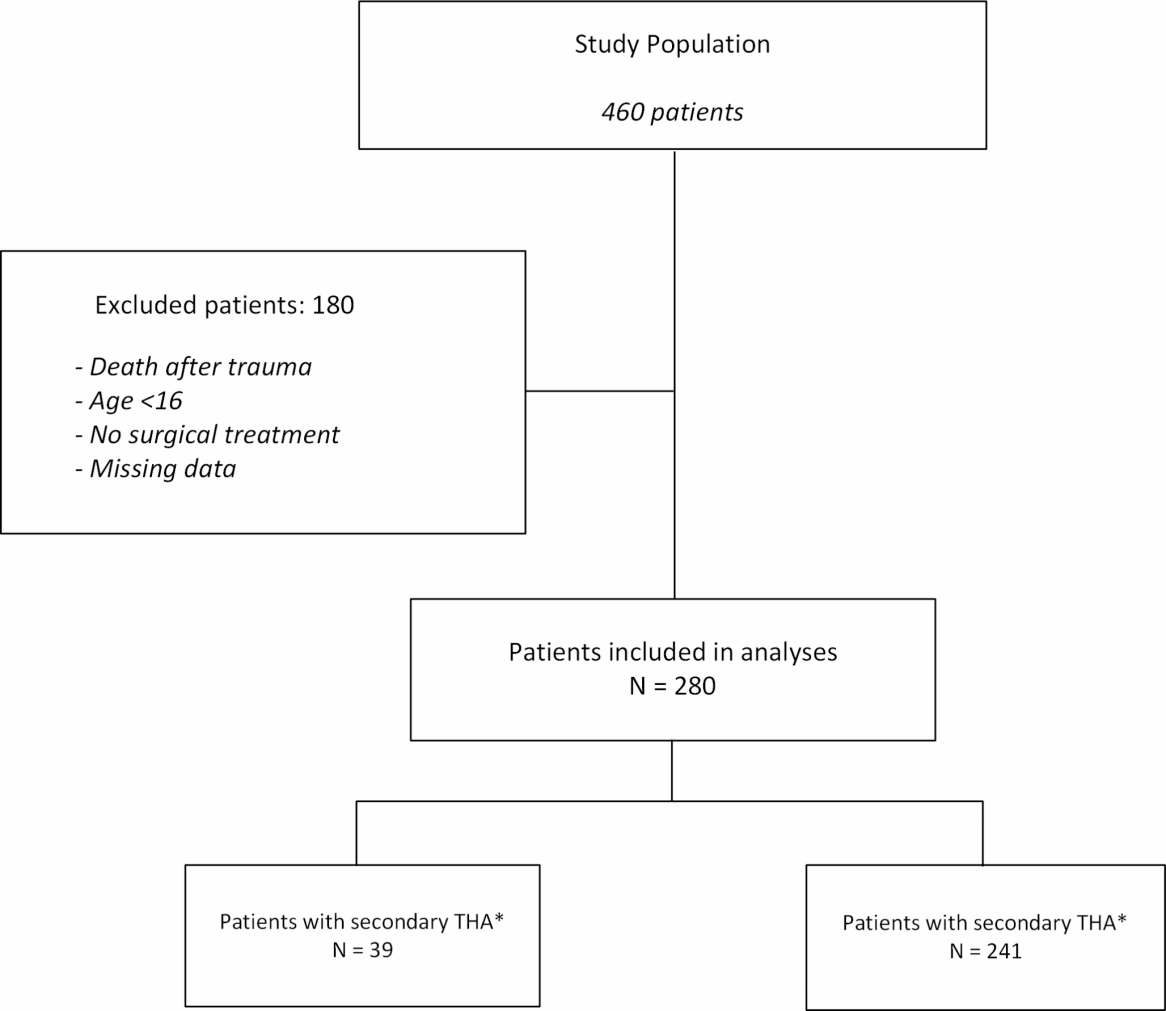
Flowchart of inclusion and exclusion of patients

### Statistical analysis

Descriptive analysis was performed to compare demographic and clinical characteristics of patients with or without secondary THA. For categorial variables a Fishers’ exact test was used, whereas for continuous variables an independent t-test was used. At last, a multivariate logistic regression model was used to control for confounding among predictive variables. Variables found with a p-value of < 0.1 in bivariate analysis were included into the logistic regression model. Means are presented with standard deviation (SD).

Data was analysed using SPSS 25.0 (Chicago, IL) for Windows. A p-value of less than 0.05 was considered statistically significant throughout the study.

## Results

Out of a total of 460 patients who were treated for acetabular fractures between 2014 and 2021, 280 patients were eligible for this study. 180 patients were excluded (death after trauma (*n* = 35), non-operative treatment (*n* = 139), age below 16, (*n* = 1) or lost to follow-up (*n* = 5)) (Fig. 1). Patient and fracture characteristics are listed in Table 1. Average patient age was 49.5 years (SD ± 17.2 years) and 77.5% of the patients were male. Posterior wall fractures (*n* = 48) and associated both column fractures (*n* = 48) were most frequent and accounted for 34% of all fractures. At initial presentation to the hospital posterior dislocation of the femoral head was seen in 37 patients (13.2%), cartilage lesion of the femoral head was seen in 11 patients (3.9%) and fracture of the femoral head in six patients (2.1%). Mean time from injury to initial surgical treatment was 5.3 days (SD ± 8.7 days). Seven patients (2.5%) received surgical treatment two weeks after trauma due to concomitant injury and their physical condition after trauma. Mean follow up was 80.4 months (SD ± 37.3 months). The overall rate of conversion to THA was 13.9% (39/280) with a mean time from initial surgery to conversion of 2.2 years (SD ± 2.1 years).

Postoperative radiological follow up showed that avascular necrosis of the femoral head (AVN) was present in 7.7% (*n* = 3) of patients converted to THA. Protrusion of the femoral head was seen in 6.5% (*n* = 2) of patients with conversion to THA. Deep infection leading to a Girdlestone procedure followed by reimplantation of a THA in a second stage was seen in 12.8% (*n* = 5) of patients. The remaining 74.4% (*n* = 29) of patients that received early conversion to THA had secondary osteoarthritis of the hip joint.

Bivariate analysis was performed to compare patients converted to THA and patients not converted to THA (non-THA) after initial ORIF-treatment (Table 1). Age at time of trauma was higher in the THA group (THA mean 58.8 years (SD ± 14.1 years) vs. non-THA mean 48.0 years (SD ± 17.2 years); *p* = 0.027). Residual displacement after reduction (> 2 mm) was more common in the THA group (THA 72.2% (*n* = 85) vs. non-THA 37.8% (*n* = 26); *p* < 0.001). Three facture patterns differed significantly between THA and non-THA groups (posterior wall (THA 5.1% (*n* = 2) vs. non-THA 19.1% (*n* = 39); *p* = 0.032), T-shape (THA 15.4% (*n* = 6) vs. non-THA 3.3% (*n* = 8); *p* = 0.001), associated both columns (THA 28.2% (*n* = 11) vs. non-THA 15.5% (*n* = 37) (*p* = 0.048)).

There was no difference between the two groups in risk factors regarding gender (male) (THA 76.9% (*n* = 30) vs. non-THA 77.6% (*n* = 187); *p* = 0.926) and the time between injury and initial surgery (THA mean 5.52 days (SD ± 9.3 days) vs. non-THA mean 3.97 days (SD ± 3.1 days); *p* = 0.484) (Table 1). Also, posterior luxation of the femoral head (THA 15.4% (*n* = 6) vs. non-THA 12.8% (*n* = 187) (*p* = 0.750), cartilage lesion of the femoral head (THA 7.7% (*n* = 3) vs. non-THA 3.3% (*n* = 8) (*p* = 0.219) and fractures of the femoral head (THA 0.0% (*n* = 0) vs. non-THA 2.5% (*n* = 6) (*p* = 0.307) did not significantly differ between THA and non-THA group.

**Table 1:** Baseline patient demographic and clinical characteristics

	Total (*N* = 280)	THA (*N* = 39)	Non-THA (*N* = 241)	*P*– value*
**Baseline characteristics**				
Age, mean (IQR)	49.5 (23.5–75.5)	58.8 (42.8–74.8)	48.0 (21.0–75.0)	0.027
Sex, Male (%)	217 (77.5)	30 (76.9)	187 (77.6)	0.926
**Letournel fracture classification**,** No. (%)**				
Anterior Column Anterior Wall Posterior Column Posterior Wall Transverse T-shape Transverse + Posterior wall Posterior column + Posterior wall Anterior wall + Posterior Hemi-transverse Associated both Column Time from injury to initial surgery in days, mean (IQR) Degree of reduction (> 2 mm)	41 (14.6) 21 (7.5) 4 (1.4) 48 (17.1) 9 (3.2) 14 (5.0) 30 (10,7) 20 (7.1) 45 (16.1) 48 (17.1) 5.31 (0.3–10.3) 111 (53.6)	2 (5.1) 0 (0.0) 1 (2.6) 2 (5.1) 1 (2.6) 6 (15.4) 4 (10.3) 4 (10.3) 8 (20.5) 11 (28.2) 3.97 (-3.97-9.97) 26 (72.2)	39 (16.2) 21 (8.7) 3 (1.2) 46 (19.1) 8 (3.3) 8 (3.3) 26 (10.8) 16 (6.6) 37 (15.4) 37 (15.4) 5.52 (0.52–10.52) 85 (37.8)	0.070 0.055 0.519 0.032 0.804 0.001 0.921 0.416 0.416 0.048 0.484 <0.001

* Comparing THA group with non-THA group. Data are mean (IQR), n(%). THA = Total Hip Arthroplasty. AO/OTA =. Arbeitsgemeinschaft für Osteosynthesefragen / Orthopedic Trauma Association. P-values are calculated between THA and No THA groups

Variables found in bivariate analysis that showed a difference between THA and non-THA group with an p-value of < 0.1 were included in a multivariate logistic regression model. Variables included in the logistic regression model were age at time of trauma (*p* = 0.027), degree of reduction (> 2 mm), anterior column fracture patterns (*p* = 0.070), posterior wall fracture patterns (*p* = 0.032), t-shape fracture patterns (*p* = 0.001) and associated both columns fracture patterns (*p* = 0.048). Fractures involving the anterior wall were excluded from the logistic regression model since there were no patients receiving early conversion to THA in this group. Multivariate logistic regression showed that age at time of accident (*p* = 0.001), residual displacement after reduction (> 2 mm) (*p* = 0.002) and t-shape fracture patterns (*p* = 0.003) had a significant increased risk of conversion to THA (Table 2). The risk of conversion to THA increased with 4.7% per gained year of age (*p* = 0.001). The presence of posterior wall or associated both column fracture patterns was not statistically significant (*p* = 0.497 and *p* = 0.264 respectively) (Table 2).

**Table 2:** Multivariate logistic regression model of predictors of early conversion to THA

	Odds Ratio	Lower 95% CI	Upper 95% CI	*P*– value*
Age at time of trauma Residual displacement (> 2 mm)	1.047 3.728	1.019 1.628	1.076 8.537	0.001 0.002
**Fracture patterns**				
Anterior column Posterior wall T-shape Associated both column fractures	0.415 1.738 7.491 1.830	0.087 0.353 1.948 0.746	1.972 8.569 28.805 4.487	0.269 0.497 0.003 0.187

The mean time from initial surgery to conversion to THA was the shortest in fractures involving both posterior wall and posterior column with a mean time of 13.8 months (SD ± 8.8 months). The longest mean time from initial surgery to conversion to THA was found in fractures involving the posterior wall with a mean of 53.0 months (SD ± 18.4 months) (Table 3).

**Table 3:** Time from initial surgery to conversion to THA

	Time from initial surgery to conversion to THA in months, mean (SD)
**Fracture patterns**	
Anterior column Posterior column Posterior wall Transverse T-shape Transverse + posterior wall Posterior wall + posterior column Anterior wall + posterior Hemi-transverse Associated both columns	29.5 (± 33.2) 26.0 (n.a.*) 53.0 (± 18.4) 14.0 (n.a.*) 26.8 (± 25.2) 20.7 (± 4.1) 13.8 (± 8.8) 44.6 (± 37.1) 31.7 (1 ± 7.4)

N.a.= not available. *= no standard deviation available due to low number of cases

## Discussion

In this retrospective cohort study the overall rate of conversion to THA after surgically treated acetabular fractures was 13.9%. T-shape fracture patterns were found to be associated with an increased risk of early conversion to THA. Other fracture patterns classified by Letournel were not identified as independent risk factors. However, bivariate analysis suggested that fractures involving the posterior wall and associated both column fractures were associated with a higher incidence of early conversion to THA, but multivariate analysis did not prove these fractures to be independent risk factors in this study. Furthermore, higher age at time of trauma and poor degree of reduction were associated with a higher risk of early conversion to THA.

The conversion rate to THA found in this study was in line with rates found in existing literature, 13.9% vs. 8.5–30.95% [1, 3, 10, 11]. The main reason for conversion to THA is post-traumatic osteoarthritis. In literature reported risk factors for post traumatic osteoarthritis are mainly higher age (> 40 years), quality of reduction (Matta’s criteria) and AVN which is seen in 5.6% of patients after femoral head trauma [3, 11].

Ziran et al. [11] performed a review of available literature to identify independent risk factors for conversion to THA after surgically treated acetabular fractures. They found that the presence of fractures involving the posterior wall increased the risk of conversion to THA. According to Firoozabadi et al. [10] fractures involving the posterior wall are the most frequent fractures overall and are known to have a conversion rate to THA of 16.9% [10]. However, in this study both column fractures were as common as posterior wall fractures (17.1% for both) and the conversion rate to THA for fractures involving the posterior wall was 4.7% (2/48). Moreover, we did not find a statistically significant increased risk of conversion to THA for fractures involving the posterior wall. Briffa et al. [1] found that fractures involving the posterior column are associated with poor outcomes. However, in our study bivariate analysis showed that fractures involving the posterior column are not a risk factor for conversion to THA. Surprisingly, no significant differences in conversion to THA were found in patients with femoral head fractures or cartilage defects of the femoral head. This may be biased by the fact that femoral head fractures are relatively rare, and that cartilage defects are not seen on conventional imaging or CT. Therefore, cartilage defects were only noted if they were visualized during surgery. This could have led to an under-reporting of such lesions.

In this study we found that higher age is an independent risk factor for conversion to THA. Ziran et al. [11] found that age > 40 years was an independent risk factor for early conversion to THA. Rollmann et al. [24] found that the risk of conversion to THA increased with 6% per year of higher age. In this study the mean age at time of trauma was 49.5 years (SD ± 17.2 years). This is similar when compared to the mean ages at time of trauma found by Firoozabadi et al. (43.7 years) and Rollmann et al. (51.9 years (non-THA group) & 57.1 years (THA group)) [10, 24].

Poor fracture reduction was also found to be a risk factor in previous literature according to Ziran et al. [11]. Verbeek et al. [25] further specified that residual gap (> 5 mm) was associated with an increased risk of early conversion to THA. Briffa et al. [1] found that t-shaped fracture patterns were associated with difficult reduction and poor outcomes. Trouwborst et al. [26] found that CT based fracture displacement of < 2 mm had limited risk in conversion to THA in non-operatively treated acetabular fractures. Step-off ≥ 2 mm and age > 60 years were found to be predictors for conversion to THA.

Delay from time of injury to initial surgery of more than 15 days and more than 5 days for elementary and associated fracture patterns respectively was associated with an increased risk of conversion to THA according to Ziran et al. [11]. In this study analysis showed that delay to initial surgery did not differ between the THA and non-THA group and was therefore not included in the logistic regression model.

Primary THA as treatment for acute acetabular fractures is of growing interest. However, data is limited and variable [19, 20]. De Bellis et al. [19] found that in elderly patients with acetabular fractures and poor bone quality, combined acetabular and femoral neck fractures or pathological fractures primary THA is as successful as delayed THA. Iqbal et al. [22] found that primary THA for complex acetabular fractures in selected elderly patients resulted in favourable Harris Hip Scores (HHS). Thirty-seven patients (78%) who underwent primary THA for complex acetabular fractures had excellent outcomes as per HHS. Jauregui et al. [20] describes higher overall incidence of femoral head dislocation and heterotopic ossification after primary THA for acetabular fractures compared to THA in a non-traumatic setting. There is no literature available that specifies outcomes of primary THA for T-shaped fracture patterns. Manson et al. [27] found that in T-type fracture patterns, bone structures that are essential for acetabular component stability for THA are disrupted. A combination of ORIF with THA could restore acetabular component stability. With growing knowledge about which risk factors contribute to conversion to THA, more research should be done to investigate whether primary THA is a successful treatment option for selected acetabular fractures.

Sample size and the monocentre setup are the main limitations of our study. The sample size needed for a power of 80% at a significance level of 95% for operatively treated acetabular fractures and conversion to THA can be estimated from Tannast et al. [6]. A total of 254.341 acetabular fractures would be needed to analyse the different types adjusted for the prevalence of each type. Since acetabular fractures are relatively rare our study population is limited. Collaboration between hospitals in future research to increase sample size could be done to further support existing literature.

Another limitation of this study is that due to some high-risk fracture patterns being rare, such as the T-shape fracture. Surgeons might be less experienced treating these fracture types. Larger volumes of rare fracture types would be needed to distinguish risk of conversion solely due to fracture type or due to lack of experience by the surgeon.

Despite this study had a retrospective setup, there was limited recall bias. Data was prospectively collected in our electronic database and processed immediately during treatment and further follow up. A few patients were lost to follow up and therefore not included in this study.

## Conclusion

In conclusion, this study showed that 13.9% of patients with operatively treated displaced acetabular fractures needed conversion to total hip arthroplasty. Mean time to conversion was 2.2 years (SD ± 2.1 years) after ORIF. The risk of early conversion to THA increased with the presence of T-shaped fracture patterns, higher age at time of trauma and poor degree of reduction. Other fracture patterns classified according to Letournel were not associated with an increased risk of conversion to THA. For patients with T-shaped acetabular fracture patterns, treatment options should be evaluated and the risk of early conversion to THA should be discussed accordingly. Also, excellent degree of reduction should be pursued intra-operatively for risk reduction of early conversion to THA. With the independent risk factors found in this study, surgeons should have a better understanding of the pre-operative risk of conversion to THA following ORIF after an acetabular fracture.

## Data Availability

No datasets were generated or analysed during the current study.
